# Powering and Fabrication of Small-Scale Robotics Systems

**DOI:** 10.1007/s43154-021-00066-1

**Published:** 2021-10-07

**Authors:** Salvador Pané, Pedro Wendel-Garcia, Yonca Belce, Xiang-Zhong Chen, Josep Puigmartí-Luis

**Affiliations:** 1 Multi-Scale Robotics Lab (MSRL), Institute of Robotics and Intelligent Systems (IRIS), ETH Zurich, CH-8092 Zurich, Switzerland; 2 Institute of Intensive Care Medicine, University Hospital of Zürich, Zürich, Switzerland; 3 Departament de Ciència Dels Materials I Química Física, Institut de Química Teòrica I Computacional, 08028 Barcelona, Spain

**Keywords:** Mobile small-scale robotics, Micro- and nanoscale propulsion, Micro- and nanofabrication

## Abstract

**Purpose of Review:**

The increasing number of contributions in the field of small-scale robotics is significantly associated with the progress in material science and process engineering during the last half century. With the objective of integrating the most optimal materials for the propulsion of these motile micro- and nanosystems, several manufacturing strategies have been adopted or specifically developed. This brief review covers some recent advances in materials and fabrication of small-scale robots with a focus on the materials serving as components for their motion and actuation.

**Recent Findings:**

Integration of a wealth of materials is now possible in several micro- and nanorobotic designs owing to the advances in micro- and nanofabrication and chemical synthesis. Regarding light-driven swimmers, novel photocatalytic materials and deformable liquid crystal elastomers have been recently reported. Acoustic swimmers are also gaining attention, with several prominent examples of acoustic bubble-based 3D swimmers being recently reported. Magnetic micro- and nanorobots are increasingly investigated for their prospective use in biomedical applications. The adoption of different materials and novel fabrication strategies based on 3D printing, template-assisted electrodeposition, or electrospinning is briefly discussed.

**Summary:**

A brief review on fabrication and powering of small-scale robotics is presented. First, a concise introduction to the world of small-scale robotics and their propulsion by means of magnetic fields, ultrasound, and light is provided. Recent examples of materials and fabrication methodologies for the realization of these devices follow thereafter.

## Introduction

Micro- and nanorobots are untethered small-scale devices that display the ability of motion in fluids when they are stimulated by means of external sources of energy such as magnetic or electric fields, ultrasound, light, or combinations thereof [1–3, 4•, 5]. Small-scale robots belong to the micro- and nanomotors’ family. However, in contrast with other small-scale motors (mainly chemically propelled swimmers), micro- and nanorobots display both controllable speeds (including on/off motion) and directionality [6–9]. Hence, this review will not deal with chemically propelled micro- and nanomotors, their propulsion mechanisms, and the materials used for their propulsion.

Small-scale robotics offer the promise of miniaturized mobile platforms for realizing tasks such as targeted therapeutic delivery, microsurgery, localized diagnosis, on-the-fly chemistry, or environmental cleaning [1, 3, 5, 10, 11]. While research in small-scale robotics is a relatively young area of research, in the past few years, efforts have been increasingly moving from fundamental studies to practical applications. This becomes especially apparent in the area of biomedical micro- and nanorobotics, where we have recently seen considerable research of these devices in in vivo models [12–16]. The swift progress in micro- and nanorobotics during the last two decades has been chiefly catalyzed by developments in material science and micro- and nanofabrication. Control over matter, shape, and assembly at micro- and nanoscales can now be accomplished by an extensive variety of methods such as 2D- and 3D-lithography [17], atomic layer deposition [18], template-assisted electrochemical processing [19], microfluidics [20, 21], or 3D (bio)printing [22], among others. The most desired approaches for manufacturing micro- and nanorobots are those that enable batch production, are compatible with other fabrication approaches, and allow for the integration or combination of a wide variety of materials. Additionally, the addition of a material should not impact the properties of other components. For example, the component of a robot that enables light-triggered motion should not be shadowed by integrating a functional material that would interfere by absorbing light, thus impairing the motion of the robot.

In the present review, we give a brief overview on the means for providing motion to small-scale robots using magnetic fields, ultrasound, and light as well as their propulsion mechanisms, with a major focus on the materials and designs that enable the locomotion of the robots.

## Propulsion of Micro- and Nanoscale Robots

Essentially, two types of forces exist on a system that moves in a fluid: inertial forces and viscous forces, the ratio of which is known as the Reynolds number (Re). Viscous forces are surface-related, while inertial forces are proportional to weight. Because at small scales, that is, at low Re, viscous forces are more relevant to those related to inertia, the mechanisms that are used for macroscale propulsion do not function at the micro- and nanorealms [23–25]. Illustratively, the mechanism that enables a scallop to swim, which is by opening and closing its hinged shells, would not work at low Reynolds number (at least in Newtonian fluids). In other words, a micro- or a nanoscopic scallop would not be able to move from its original position, and no net translation would be attained. This phenomenon is reflected in the Scallop’s theorem [26–28], which states that in order to achieve net translation, a non-reciprocal stroke or deformation is necessary. Nature has engineered machinery to overcome the challenge of motion at small scales by providing tools and mechanisms such as rotating chiral appendages or beating oars. For instance, certain bacteria propel by rotating a bundle of helical flagella [14, 29, 30]. Sperm cells swim by swinging their tail to move through the highly viscous seminal fluid [15, 29, 31]. Other mechanisms for motion at small scales exploit friction on surfaces. For example, cells such as keratocytes or neutrophils roll on tissues to translocate[32].

While challenges in small-scale propulsion still exist specially in complex biological fluids, researchers have demonstrated several strategies to provide locomotion to micro- and nanoarchitectures. In the area of small-scale robotics, the use of external sources of energy such as light, ultrasound, electric fields, magnetic fields, or combinations of these has been proposed [2, 6, 10, 33, 34••]. The mechanism used for propulsion not only determines the specific materials, which have to be specifically reactive to the external input of energy, but also the optimal fabrication route for the construction of the device. In the following, we briefly comment on each type of locomotion strategy.

### Light-Driven Small-Scale Robots

**Fig. 1:**
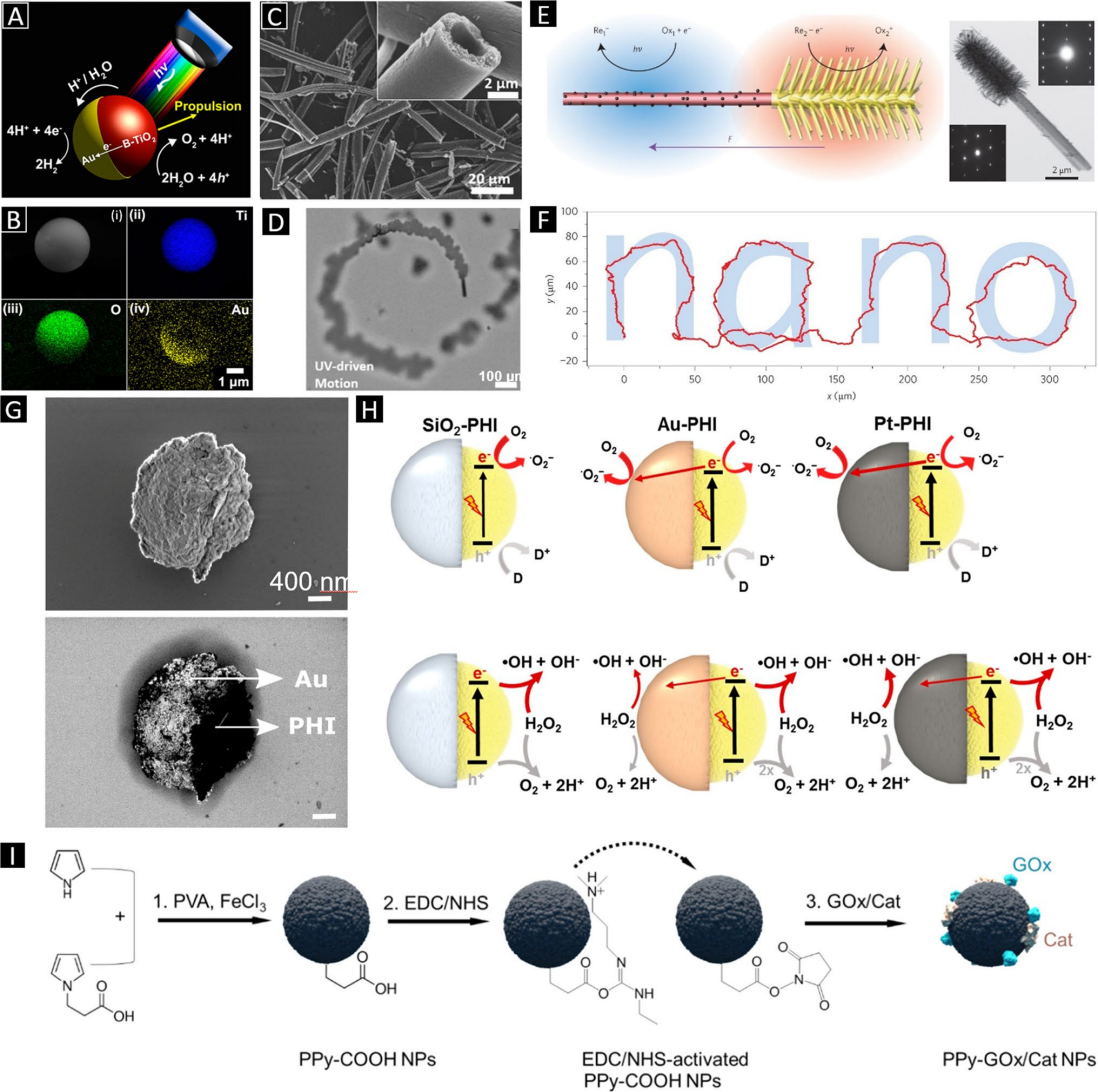
**A** A schematic illustration of the propulsion mechanism of Au/B-TiO_2_ Janus micromotors. **B** An SEM image and corresponding EDX image of a Au/B-TiO_2_ Janus micromotor: (i) The SEM image and EDX mapping of (ii) titanium, (iii) oxygen, and (iv) gold, respectively (Fig. 1a and b: Adapted from [36]. Copyright © 2017 American Chemical Society.). **C** SEM images of TiO_2_ microtubes. **D** Light-controlled motion of a TiO_2_ microengine in H_2_O_2_ solution (Fig. 1c and d: Adapted from [38]. Copyright © 2015 WILEY–VCH Verlag GmbH & Co. KGaA, Weinheim). **E** A schematic illustration and a TEM of a nanotree. **F** The trajectory of a nanotree navigated by light showing “nano”. (Fig. 1E and F: Adapted from [39]. Copyright © 2016 Springer Nature.) **G** SEM images of Janus particles taken with in-lens mode and back-scattered electron mode. **H** Illustrations showing different propulsion mechanisms in water and H_2_O_2_. (Fig. 1g and h: Adapted from [77]. Copyright © 2020 National Academy of Sciences.) **I** Schematic representation of the sequent process of nanoparticle preparation, activation, and enzyme immobilization. (Adapted from [43]. Copyright © 2021 American Chemical Society.)

Propulsion of micro- and nanostructures using light has been achieved in different ways, for instance, exploiting the photocatalytic features of certain materials. When a photocatalytic material is exposed to light of a particular wavelength, separation of electrons and holes takes place [35]. Electrons and holes, in turn, can react with water or other available redox compounds in a surrounding electrolyte, generating different types of radical species. Yet, to be able to achieve an optimal propulsion, usually a material that can act as acceptor of electrons or holes is required to avoid the recombination of these within the photocatalytic material. In the example given (Fig. 1(A) and (B)), a photocatalytic swimmer is presented [36]. The swimmer is a Janus microsystem comprising a black titania sphere, which acts as the photocatalytic material, and a gold hemispherical coating serving as an electron acceptor. Black titania is able to absorb light in both the visible and the ultraviolet region of the spectrum of light. Hence, when it is subject to light, electrons and holes are generated. Electrons travel to the gold region, thus generating an in-built electric field. Electrons on the gold side react with protons in water, while water is oxidized at the black titania surface, overall generating a proton gradient that causes the Janus system to propel by self-electrophoresis. Other photocatalytic systems have been designed to be propelled by osmotic pressure. Pine and co-workers have shown that hematite peanut particles in solutions containing hydrogen peroxide (H_2_O_2_) can swim when subject to blue light [37]. Because of the photocatalytic decomposition of H_2_O_2_ mediated by these particles, a chemical gradient is generated at the particle-electrolyte interface. As this gradient is symmetrically distributed around the surface of the particles, those remain in their positions. Yet, a roughening surface treatment of the particles enables an asymmetric chemical gradient distribution that causes the self-propulsion of the particles. The authors also demonstrate cargo transportation using their photosensitive microstructures. Bubble propulsion is also another possible mechanism for photocatalytic swimmers. Guan and co-workers have recently shown that tubular microarchitectures made of photocatalytic titania can be propelled by UV-light-induced bubbles [38]. In this case, a fuel such as hydrogen peroxide that decomposes in gas species such as molecular oxygen is necessary (Fig. 1(C) and (D)). In principle, the photocatalytic swimmers discussed in this section do not qualify as robots because their directionality is not controlled. However, it is possible to control and program the directionality and speed of photocatalytic swimmers using chemical modifications. Tang and co-workers have demonstrated that by controlling the zeta potential of a self-electrophoretic Janus nanotree (Fig. 1(E) and (F)) architecture is possible to engineer its (negative or positive) phototactic behavior [39]. The nanotrees were actuated in solutions containing diverse fuels such as H_2_O_2_ or benzoquinone/hydroquinone redox couple.

Recently, several papers have reported locomotion of swimmers by exploiting photocatalysis. The vast majority of these swimmers consist mainly of Janus particle designs, the fabrication of which usually involves a wet chemical process (and sometimes an additional thermal treatment) for synthesizing the photosensitive chassis structure followed by the physical vapor deposition (e.g., evaporation, sputtering) of the material that covers half of the chassis surface. Mainly, titanium dioxide has been used as the photoactive part, although this material, unless treated, only absorbs in the UV region of the light spectrum. Yet, some recent studies have introduced other interesting materials for photocatalytic locomotion of micro- and nanoswimmers that can be operated under visible light. For example, Jang et al. demonstrated that upon a simple thermal treatment, titania could be easily transformed in black titania, which allows for building Janus micromotors with an evaporated gold-coated hemisphere [36]. The swimmers can be propelled not only under UV illumination but also in the entire region of the visible light spectrum. Interestingly, the speed of the swimmers could be tailored by changing the light wavelength, with the swimmers being faster under UV irradiation and blue light and slower towards higher wavelengths. The swimmers were able to propel both in solutions containing hydrogen peroxide and also in pure water. Cai and co-workers have recently demonstrated that cuprous oxide filled with nitrogen-doped carbon nanotubes (N-CNTs) can be propelled under green and blue lights by reacting with non-cytotoxic glucose as a fuel [40]. The swimmers were fabricated using a wet chemistry procedure on which cuprous oxide was precipitated in the presence of N-CNTs. Lotsch and co-workers have recently reported about two-dimensional organic poly(heptazine imide) carbon nitride microparticles (Fig. 1(G)) [41]. Carbon nitrides are an interesting option as their motion capability is not significantly influenced by the ionic nature of the swimming media. Additionally, they can also be actuated under visible light. By asymmetrically irradiating the structure, photocatalytic processes are triggered, subsequently generating an ion flow around and through the particle. Figure 1(H) shows the schematics of the proposed mechanism for propulsion. The swimmers are fabricated by means of a potassium thiocyanate melt synthesis employing melamine. Escarpa and co-workers recently reported on polystyrene-based Janus micromotors with a gold hemisphere decorated with quantum dots for light propulsion [42]. The quantum dots consisted of core–shell structures with cadmium selenide cores coated with zinc sulfide. The swimmers were propelled by bubble propulsion using hydrogen peroxide as a fuel and upon irradiation with UV or visible light. Lyutakov and co-workers have recently reported on glucose-fueled sun light-driven polypyrrole-based nanoswimmers [43]. Two types of nanorobots were fabricated using a wet chemistry polymerization approach in which pyrrole and 1-(2-carboxyethyl)-pyrrole were used as monomers and mixed in a solution containing iron(III) chloride as oxidant and polyvinyl alcohol. Next, the resulting polypyrrole-based nanoparticles were decorated with glucose oxidase and catalase enzymes, which were anchored to the nanoparticles through the terminal carboxylates activated by N-(3-dimethylaminopropyl)-N′-ethylcarbodiimide hydrochloride. See Fig. 1(I) for the fabrication scheme of the swimmers.

**Fig. 2:**
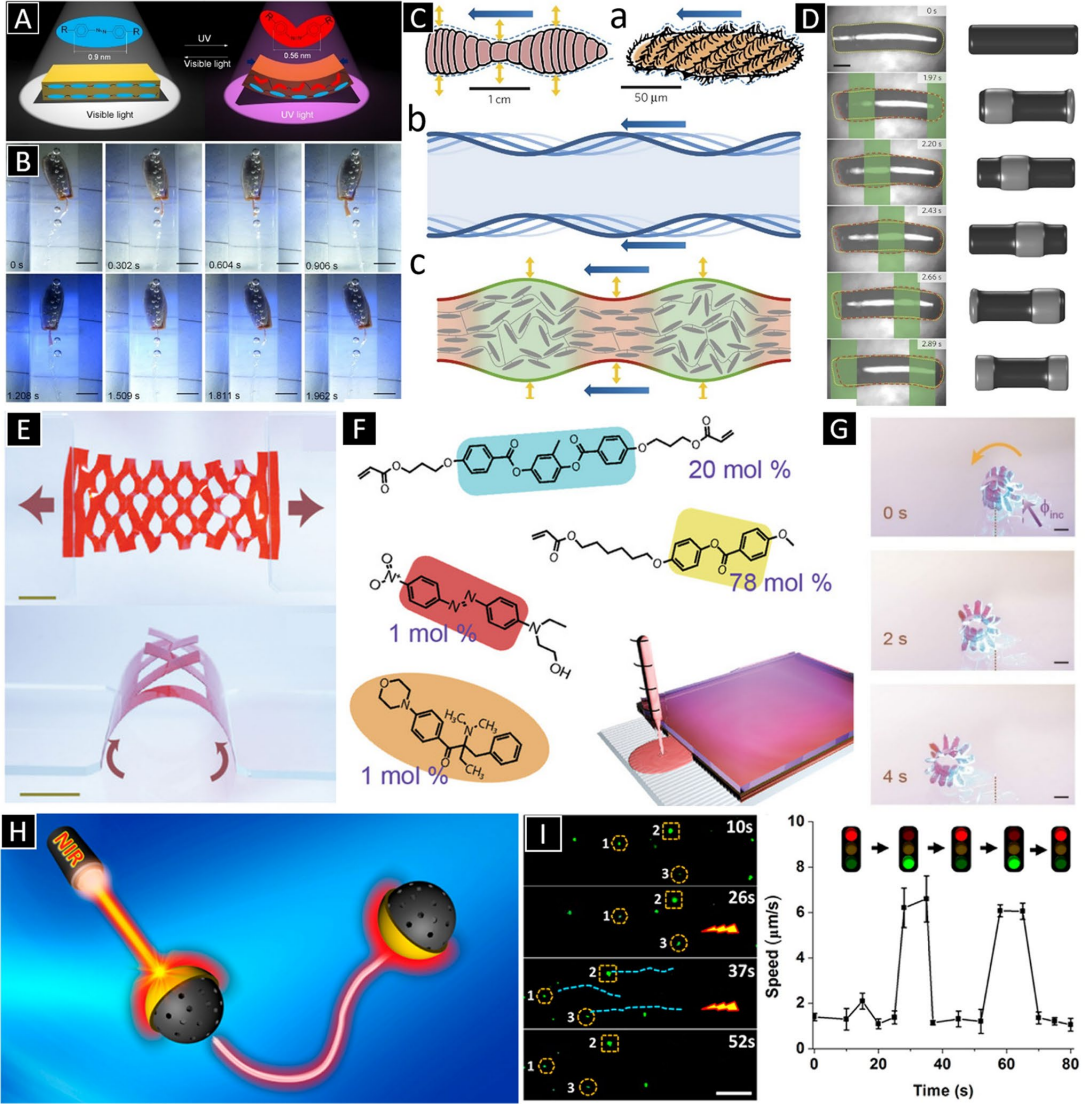
**A** A schematic illustration of transformation of azobenzene between trans and cis conformation and associated shape changing. **B** Periodic swing of the flagellum in a cycle. The scale bar is 2 mm. (Fig. 2a and b: Adapted from [44].) **C** (a) Inspiration from peristaltic locomotion of a worm and a ciliate. (b) Abstraction of the concept of traveling waves as a general locomotion principle and (c) its realization using soft active materials. **D** Images showing the deformation of a light driven microrobot under a periodic light pattern scanned from left to right. Scale bar, 200 μm. Corresponding simulations of the behavior of the microrobot is shown on the **left**. (Fig. 2C and D: Adapted from [45]. Copyright © 2016 Springer Nature.) **E** Images of liquid crystal network-based kirigami structure (Scale bars 5 mm). **F** Composition of the precursor for synthesizing the LCE. **G** A kirigami structure is actuated by the visible light (Scale bars 2 mm). (Fig. 2E, F, and G: Adapted from [47]. Copyright © 2020 John Wiley and Sons.) **H** A schematic illustration of near-infrared light-powered Janus mesoporous silica nanomotors. **I** Time-lapsed images of the “on/off” motion of a silica nanomotor triggered by NIR laser (left panel) and the corresponding speed–time relationship (right panel). Scale bar = 20 μm. (Fig. 2H and I: Adapted from [48]. Copyright © 2016 American Chemical Society.)

Another strategy to propel structures with light consists of using materials that deform or morph under irradiation. An example of these materials is liquid crystal elastomers (LCEs) containing photoisomerizable molecules. Liu and co-workers, for example, demonstrated soft robotic structures made of polymers embedding azobenzene chromophores [44]. Upon a UV-light irradiation, the azobenzene undergoes a trans–cis isomerization, which macroscopically results in a deformation (Fig. 2(A) and (B)). The original shape is recovered by exposing the structure to visible light [44], which brings the molecules back to their trans conformation. Using these materials, the researchers realized a microrobot composed of a LCE-based flagellum and a head comprising an LCE-based gripper. The robot was able to perform wave-like swinging when exposing light to the flagellum. Additionally, pickup of cargo with the light-driven gripper and transportation was demonstrated. Recently, Fischer and co-workers demonstrated the fabrication of cylindrical robots made of azodyes-containing LCEs [45]. By means of structured light, the soft robotic structures were able to swim under a periodic light pattern which was scanned through the robotic structure from one head to the other. The robot was able to mimic the propulsion mechanism of ciliate microorganisms, which swim by a coordinated cilia motion that results into traveling metachronal waves (Fig. 2(C) and (D)). For an extensive review on light-driven swimmers, the reader is referred to the reviews [33, 46].

Swimmers and robotics structures built upon liquid crystal polymer networks are difficult to be processed at the micro- and nanoscale. Usually, these materials are prepared in bulk solutions, then molded or deposited as films by spin coating, and finally photocured. Scalpels or laser are then used to cut the structures. Figure 2(E) shows a liquid crystal network-based kirigami structures that have the ability of changing shape or locomote upon light irradiation reported by Priimagi and co-workers [47]. The liquid polymer network was fabricated using a mixture containing the monomer 4-methoxybenzoic acid 4-(6-acryloyloxyhexyloxy)phenyl ester, a cross-linker, the light-responsible azo molecule, and a photoinitiator (Fig. 2(F)). The mixture containing the polymer was then infiltrated between two chemically modified glass slides with spacers and then photopolymerized. The resulting films were then cut by laser to provide the desired 2D pattern for the kirigami. The cut pattern was then rolled up around a tube structure to induce a tubular shape. Figure 2(G) shows the rolling motion of the kirigami structure under light activation.

The phototermal effect has also been used for the manipulation of micro- and nanostructures. By creating a thermal gradient around a small-scale object, this can be propelled as a consequence of thermophoresis or Soret effect, in short, by thermal diffusion of the fluid. In order to achieve opto-thermophoresis, usually the swimmer should exhibit an asymmetric distribution of its components, that is, the photothermally sensitive material should be arranged asymmetrically or in a specific location in the swimmer architecture. For example, Janus architectures have been widely employed to demonstrated photothermally induced propulsion. In Fig. 2(H) and (I), an example of this kind of propulsion concept is illustrated. He and co-workers demonstrated that Janus particles consisting of mesoporous silica nanoparticles coated by a light-absorbing gold hemisphere can swim due to self-thermophoresis when they are subject to near-infrared (NIR) light [48]. The authors demonstrated that the nanoswimmers propulsion, including their on/off motion, could be tuned by the NIR laser. For an extended explanation of the working principles of optically induced thermorphoretic propulsion, we derive the readers to the review by Sun and co-workers [49].

### Acoustically Driven Small-Scale Robots

**Fig. 3:**
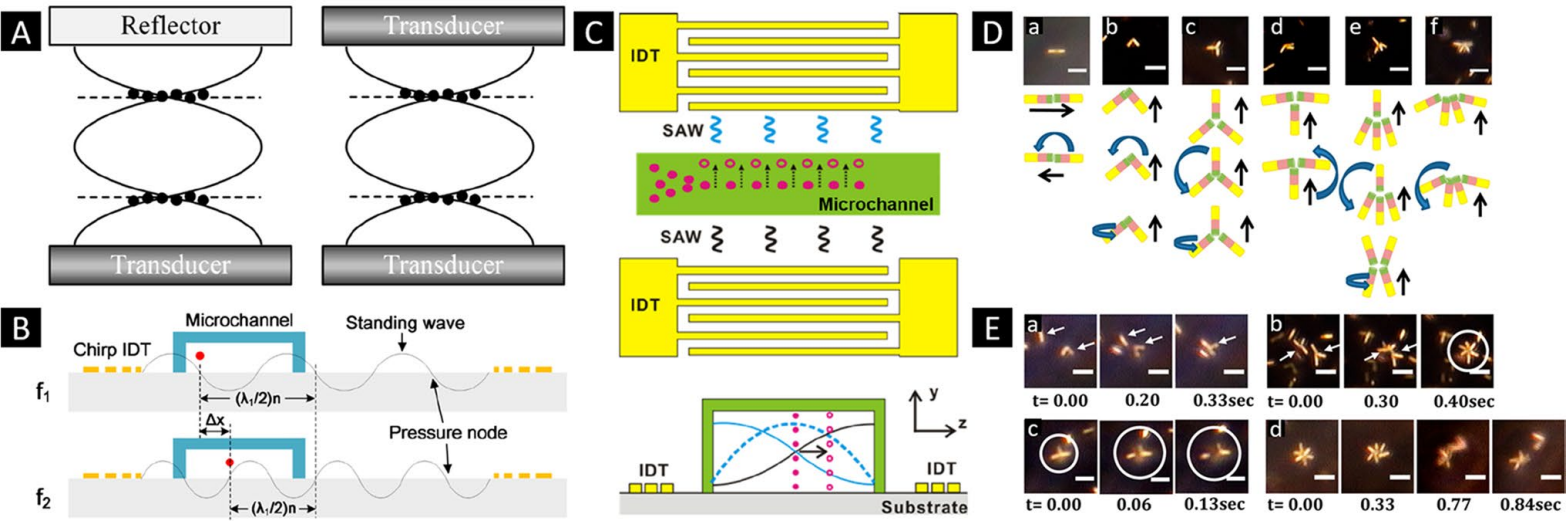
Schematic representation of a standing wave form **A** obtained by reflection or interference of two acoustic waves. **B** Illustrative image of relative phase **C** between interdigital transducers (IDTs) and transportation of particles is achieved depending on the position of the pressure node change, respectively. (Fig. 3A, B, and C: Adapted with permission [51]. Copyright © 2019 IOP Publishing Ltd.) **D** Optical micrographs and schematic images of the most common modes of motion of dimers, trimers, and tetramers of multisegmented nanorods under acoustic fields. Black and blue arrows indicate translational and rotational motions, respectively. The scale bars are 5 µm. **E** (a-c) Assembly of nanorod multimers by bimolecular collisions and (b-d) spontaneous disassembly sequences of nanorod multimers. The ultrasonic power determines the formation of dimers, trimers, or multimers. The scale bars are 5 µm. (Fig. 3D and E: Reproduced with permission [78]. Copyright © 2014 American Chemical Society.)

**Fig. 4:**
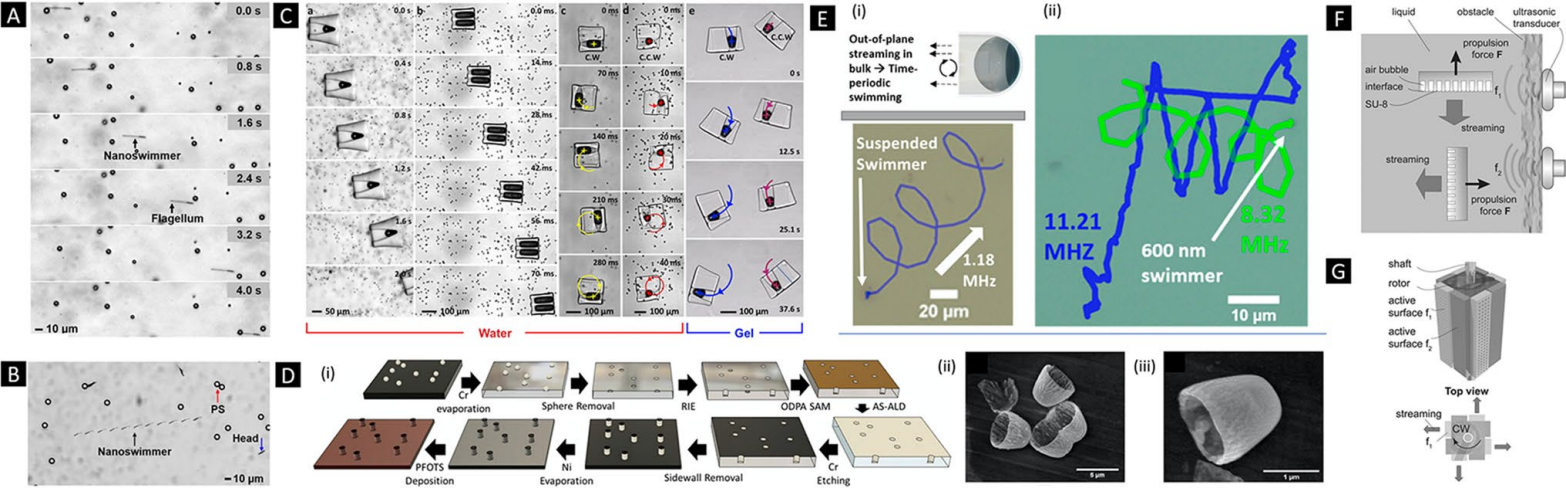
**A** Image sequence of translational motion of a flagellated nanoswimmer in traveling acoustic waves. **B** Superimposed sequences of motion of an acoustically propelled flagellated nanoswimmer in water containing 10-µm polystyrene particles. (Fig. 4A and B: Reproduced with permission [52]. Copyright © 2016 American Chemical Society.) **C** Image sequence of bubble-propelled microswimmers during translational and rotational motion through (a-d) water and (e) hydrogel. (Adapted with permission [53]. Copyright © 2015 Springer Nature.) **D** (i) Schematic representation for the wafer-based fabrication method of bubble-propelled swimmers of different scales (from macro to nanoscale). SEM images of (ii) 3-µm and (iii) 1-µm bubble swimmers. **E** Illustrative and trajectory image of a (i) microswimmer moving in a spiral path in the presence of 1.18 MHz acoustic field and (ii) a nanoswimmer changing from 2D swimming to self-spiraling. (Fig. 4D and E: Reproduced with permission [79]. Copyright © 2020 American Chemical Society.) **F** Demonstration of bubble array streaming surfaces. **G **Schematic representation of a rotary motor comprising different bubble array surfaces (the rotation is in CW). (Fig. 4F and G: Reproduced with permission [55]. Copyright © 2016 AIP Publishing.)

Ultrasound is a relatively recent approach that has been applied to propel micro- and nanorobots [50•]. Basically, standing waves, traveling waves, single beam, or arbitrary wave fields can be used to effectively manipulate small-scale objects, including biological structures [51]. In standing waves, the structures are trapped at the waves’ pressure nodes (or antinodes). The motion of the structures can be controlled by changing the nodes positions, which is achieved by modulating the resonant frequency or relative phase of the waves generated at different transducers (Fig. 3(A-C) ). Mallouk and co-workers demonstrated that by means of acoustic standing waves, bimetallic segmented nanorods not only could be propelled but also rotated, aligned, and assembled (Fig. 3(D-E)). While the use of standing waves is very useful for the propulsion of small-scale objects, its applicability in vivo is challenging because standing waves cannot be reliably established in living organisms [52]. A more robust approach for in vivo applications would be the use of traveling acoustic waves. In traveling waves, a unidirectional propulsive force is created enabling swimmers to move along the direction of the wave propagation. The propulsive force is a result from the interactions of the swimmers with the ultrasound. Additionally, compared with standing waves, the acoustic radiation forces in traveling waves are more sensitive to the size of structures. Ahmed et al. demonstrated the motion of nanowire-based swimmers consisting of a bimetallic head linked to a polypyrrole flexible tail or flagellum (Fig. 4(A)). When the swimmer is at resonance, propulsion of the nanowire by means of a propagating wave is observed. Figure 4(B) shows a sequence of the swimmer moving through water containing polystyrene microbeads under a traveling wave. Acoustic manipulation can also be achieved by acoustic streaming, which allows capturing structures by an acoustically generated streaming vortex. Based on this, Ahmed et al. demonstrated the acoustically triggered propulsion of microstructures containing a trapped bubble in an engineered microcavity [53] (Fig. 4(C)). When subject to ultrasound at the conditions of the bubble’s resonance frequency, the bubble is at its maximum oscillation amplitude. Consequently, a pair of counter-rotating vortices is generated in the surrounding fluid, creating a steady flow, known as acoustic microstreaming, which ultimately results in the propulsion of the swimmers.

There are virtually no restrictions in the type of material that could be manipulated using acoustic fields, the complexity rather lies in the design of the architecture, in other words, dissimilar density of the different parts composing the swimmer or their shape anisotropy [54]. Indeed, researchers have been able to produce composite materials consisting of different types of particles (e.g., ceramic, metallic, polymeric) organized in 1-D and 2-D arrangements by means of ultrasound [51].

Recently, Mallouk and co-workers have recently reported on a wafer-scale manufacturing approach for the fabrication of bubble-based micro- and nanoswimmers (Fig. 4(D)). The swimmers consisted of cup-shaped architectures made of polysterene. Briefly, polystyrene beads of different sizes were spin coated on wafer substrates. By means of reactive ion etching, it was possible to generate holes of different dimensions and shapes (conical or cylindrical), changing the etching pressure and plasma power. Depending on the shape and volume resonances, the trajectories of groups of swimmers could be adjusted. Figure 4(E) shows a 3-µm microrobot moving in a spiral path. Fischer and co-workers have shown that functional acoustic surfaces can be used for a multiple-degree-of-freedom wireless actuation [55]. Figure 4(F) shows the schematics of the bubble array surfaces. The surfaces were created by means of photolithography of SU-8 photocurable resin. When bubbles are at resonance, streaming of the surrounding liquid is generated at the bubble array surface, which results in a propulsion force acting on the opposite site. The surfaces can be integrated in millimeter-scale devices, such as the rotary motor shown in Fig. 4(G). He’s group has recently reported the acoustic propulsion of liquid-alloy colloidal nanorods [56]. Interestingly, the 1-µm nanorods were fabricated by means of acoustic crushing of the liquid metal in a fluid. The nanorods were manipulated using standing waves and displayed a directional motion along their long axis. The nanorods also displayed photoluminescence and were tested for their biocompatibility features with intracranial glioblastoma cells.

### Magnetically Driven Small-Scale Robots

**Fig. 5:**
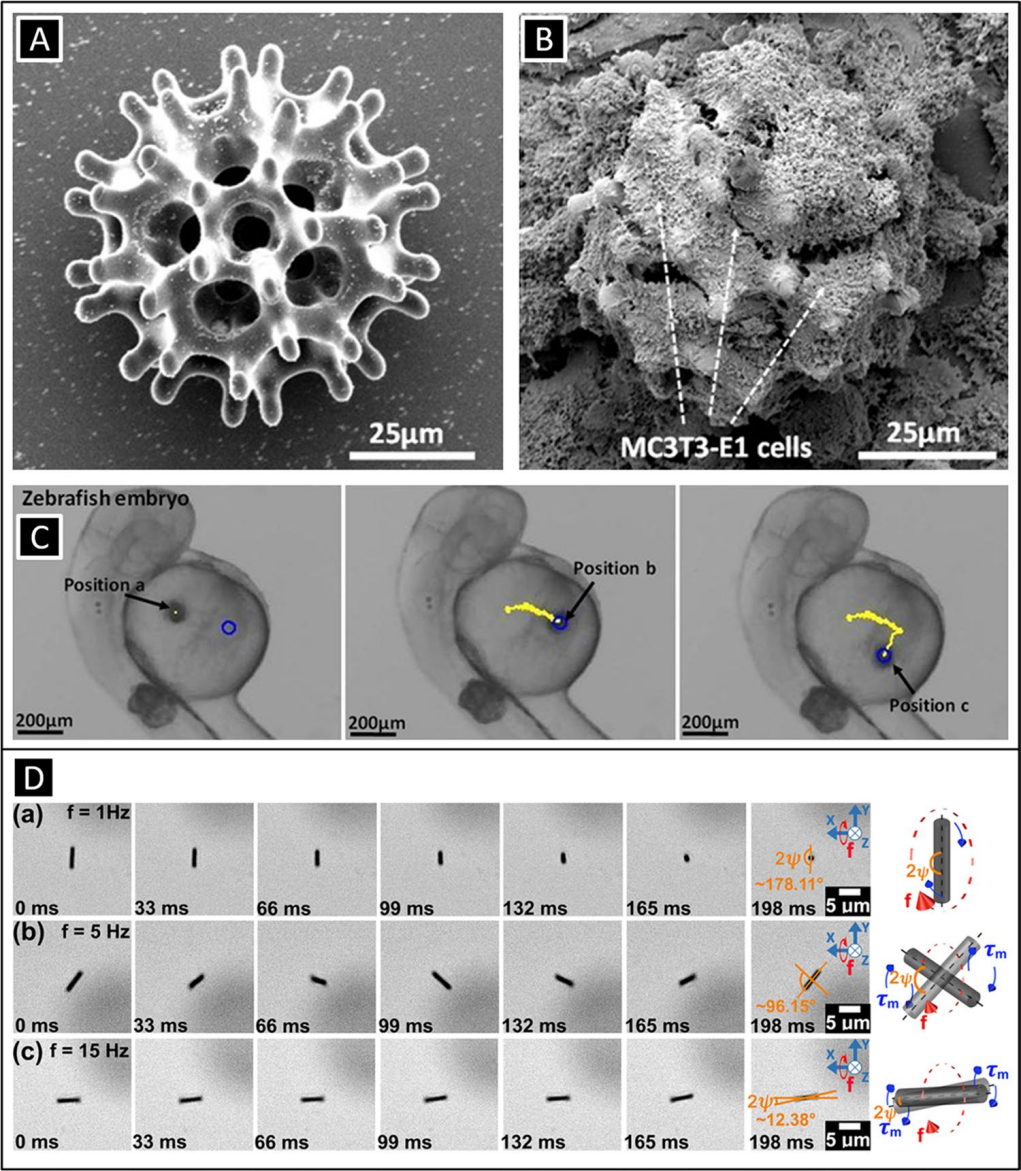
SEM characterization of a **A** burr-like 3D microrobot cultured with **B** MC3T3-E1 cells. **C** A MSC-cultured burr-like microrobot swimming through the yolk of a zebrafish embryo. (Fig. 5A, B, and C: Adapted with permission [57]. Copyright © 2018 American Association for the Advancement of Science.) **D** Locomotion mechanisms of a single hard-magnetic CoPt nanowire as a function of the frequency f of a rotating magnetic field (15 mT) in the YZ plane: (a) tumbling motion (f = 1 Hz), (b) precession (f = 5 Hz), and (c) rolling motion (f = 15 Hz). Each locomotion mechanism is represented on the right. (Adapted with permission [58] Copyright © 2018 American Chemical Society.)

Magnetic fields are arguably the most adopted form of energy for the propulsion of micro- and nanorobots. Magnetic fields can be applied in form of gradients, oscillating or rotating magnetic fields by means of magnets or electromagnetic coils. The richness in the way magnetic fields can be applied together with their biocompatible characteristics (i.e., low or null interaction with organic matter within a wide range of magnetic field and frequency values) makes this approach very attractive especially for biomedical applications. Additionally, there exists a wealth of possibilities in terms of magnetically responsive materials (diamagnetic, ferro- and ferrimagnetic, paramagnetic), which today can be constructed in very complex shapes. A relatively simple way of manipulating magnetic micro- and nanostructures consists of using gradients, which allows for exerting forces on structures. Figure 5 shows 3D-printed porous spherical microrobots carrying cells being driven by means of magnetic field gradients in vitro and in vivo inside a zebrafish embryo [57]. The robots consisted of a polymeric chassis coated with a ferromagnetic material (nickel) and titanium for biocompatibility.

**Fig. 6:**
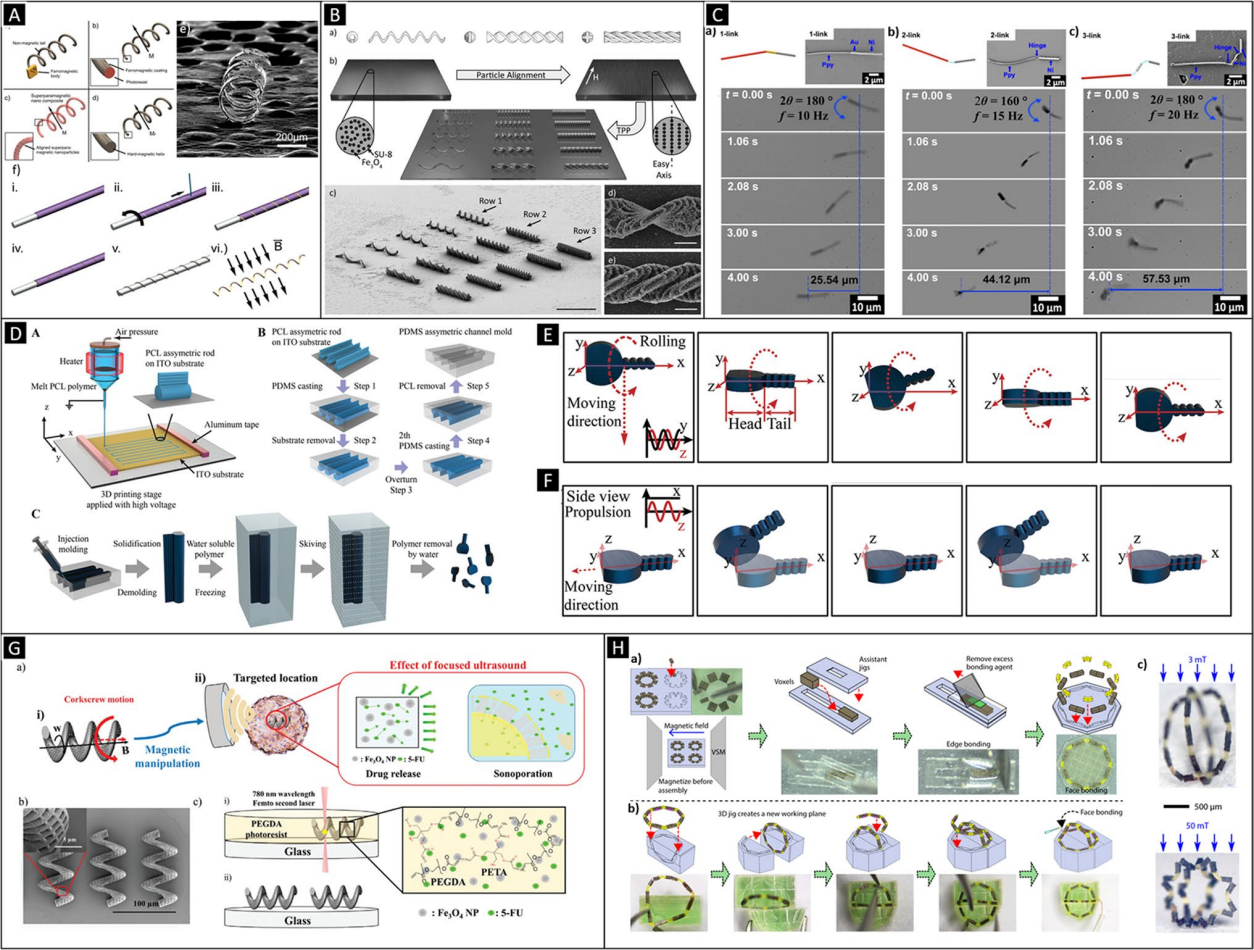
**A **Different types of helical small-scale robots made of (a) a soft-magnetic head and non-magnetic tail, (b) nonmagnetic chassis coated with a conformal ferromagnetic thin coating, (c) a superparamagnetic nanocomposite full body, (d) a hard-magnetic material full body, (e) SEM image of an electroformed semi-hard-magnetic CoPt helix, (f) fabrication sequence of helices obtained by electroforming: (i) coating of an aluminum mandrel with a polymeric insulator (ii) laser patterning of the insulating polymeric layer, (iii) electroless gold deposition through the laser-formed trenches, (iv) electrodeposition of a ferromagnetic on the gold parts, (v) dissolution of the insulating layer, and (vi) selective etching of the aluminum mandrel and perpendicular pre-magnetization of the resulting helical structure. (Adapted with permission [68]. Copyright © 2018, WILEY–VCH Verlag GmbH & Co. KGaA, Weinheim.) **B** (a) Types of magnetic nanocomposite helical microrobots (front and side view): left: helical shape; center: single twist-type shape, right: double twist-type shape. (b) Manufacturing of magnetic nanocomposite microrobots by means of two-photon polymerization. The fabrication sequence comprises spin coating (top left), magnetic field-assisted particle alignment (top right) and direct laser writing (center). (c) SEM image of the fabricated helical swimmers (Row 1), single twist-type shape (Row 2), and double twist-type shape (Row 3) (scale bar 50 μm). (d and e) Detail of single and double twist-type architectures, respectively, (scale bars: 5 μm.) (Adapted with permission [69]. Copyright © 2014 WILEY–VCH Verlag GmbH & Co. KGaA, Weinheim.) **C** Morphological and motion characterization of nanowire-based 1-, 2, and 3-link swimmers. Each swimmer displays an overall length of ~ 15.5 μm. (a) A 1-link nanoswimmer made of Ni − Au − Ppy (scheme and SEM). (b) A 2-linked nanoswimmer made of Ni − PAH/PSS − Ppy (scheme and SEM). (c) A 3-linked nanoswimmer Ni − PAH/PSS − Ni − PAH/PSS − Ppy (scheme and SEM). (Adapted with permission [70]. Copyright © 2015 American Chemical Society.) **D** Schematic manufacturing sequence of magnetic tadpole-like microrobots. (A) Schematic manufacturing sequence of polycaprolactone (PCL) asymmetric rod template, (B) polydimethylsiloxane (PDMS) asymmetric channels, and (C) PCL/Fe3O4 asymmetric microrobots. (Adapted with permission [70]. Copyright © 2015 American Chemical Society.) **E** 3D schematics of microrobot motion under rotating magnetic fields. **F** 3D schematics of the locomotion of a tadpole-like microrobot. (Figs. 6D, E, and F: Adapted with permission [73]. Copyright © 2021 The Authors. Advanced Science published by Wiley‐VCH GmbH.) **G** (a) Ultrasound-mediated drug release from a magnetically controlled porous microhelical robot: (i) corkscrew motion to a targeted area using a rotating magnetic field and (ii) application of a focused ultrasonic beam at the targeted area. (b) SEM characterization of the microrobots. (c) Fabrication of the porous helical microrobots: (i) direct laser witting laser of spin-coated polyethyleneglycoldiacrylate photoresist and (ii) resulting helical microswimmers. (Adapted with permission [74]. Copyright © 2020 Wiley‐VCH GmbH.) **H** Jig-assisted manufacturing of magnetic 3D rings with programmed magnetic sections. (a) Designs, fabrication scheme, and pictures of the assistant jigs. (b) A 3D jig assists the assembly of a ring and two half rings to generate a 3D soft robotic architecture. (c) Optical images showing the actuation and shape transformation of the 3D ring under magnetic fields. (Adapted with permission [75]. Copyright © 2021 American Association for the Advancement of Science.)

Rotating and oscillating magnetic fields have also been used to propel magnetic micro- and nanostructures. As magnetic materials exhibit an easy magnetization axis mainly induced by the shape (shape anisotropy), structures can rotate by aligning synchronously this preferential magnetization axis with the applied rotating (or oscillating) magnetic field caused by the exerted magnetic torque. Thus, by controlling the frequency of the rotating or the oscillating magnetic fields, it is possible to control the speed of the magnetic small-scale swimmers up to a certain frequency value, known as step-out frequency. Above this value, magnetic structures are not able to reorient fast enough with the magnetic fields and their speed is then reduced upon increasing further the frequency. Using rotating magnetic fields and depending on the specific geometrical shape of the swimmer, it is possible different types of locomotion. A very simple approach consists of manipulating structures such as spheres, nanowires, or more complex architectures that under the action of a rotating magnetic field can tumble or roll next to a surface. Figure 5(D) shows hard-ferromagnetic nanowires being manipulated over a substrate. The nanowires exhibit different behaviors as a function of the applied magnetic field frequency from tumbling and precession motion at low and medium frequencies, to rolling at higher frequencies, respectively [58]. With more complex structures, such as hinged nanowire-based swimmers, it is also possible to achieve helical motion. Recently, Wu et al. have shown that hinged structures consisting of a metallic tail linked to a magnetic head can swim describing helical paths under purely rotating magnetic fields [59]. The structures mimic the helical klinotactic motion of certain bacteria and cells such as spermatozoa. Rotating magnetic fields have been widely employed to study the locomotion of one of the most explored small-scale robotic architectures: helical micro- and nanorobots [60, 61]. Under rotating magnetic fields, these architectures are able to display a corkscrew mechanism that mimics the propulsion mechanism of certain flagellated microorganisms such as bacteria. Helical small-scale robots either consist of a magnetic head with a helical tail [62]; are comprised of a non-magnetic helical body conformally coated by a magnetic layer [60, 63–65]; or are made of a fully magnetic material (i.e., metal, alloy, magnetic polymer composite) [66, 67]. In the latter, careful attention must be paid to the material design. In principle, the preferential magnetization axis of a fully magnetic helical structure is along its long axis. This means that when applying a rotating magnetic field, the structure would not corkscrew but tumble. By programming the easy magnetization direction of the helical structure through its short axis, it is possible to achieve a corkscrew locomotion with fully magnetic bodies [67, 68]. Two strategies exist: (a) by pre-magnetizing a hard-magnetic helix along its short axis [68] or (b) by manufacturing helices using magnetic nanocomposites in which magnetic nanoparticles are aligned parallel to the helix short axis [69]. Figure 6(A) and (B) show different hard-magnetic and magnetic nanocomposite helical designs [68][69].

Using oscillating fields, interesting locomotion mechanisms can also be achieved. For example, Nelson, Or, Pané, and co-workers showed that by applying oscillating fields to hinged nanowire-based structures consisting of a flexible tail and a magnetic head, it is possible to emulate the swimming mechanism of eukaryotic cells [70]. Figures 6 (C) and (D) show that by increasing the number of hinges, it is possible to achieve a more defined traveling wave, which allows for a more effective propulsion of the assembly. Wang and co-workers demonstrated another interesting propulsion mechanism similar to the locomotion of fishes with similar hinged nanowire-based architectures [71]. The same team also demonstrated free-style swimming with a nanorobotic system containing two magnetic nanoarms [72].

**Fig. 7:**
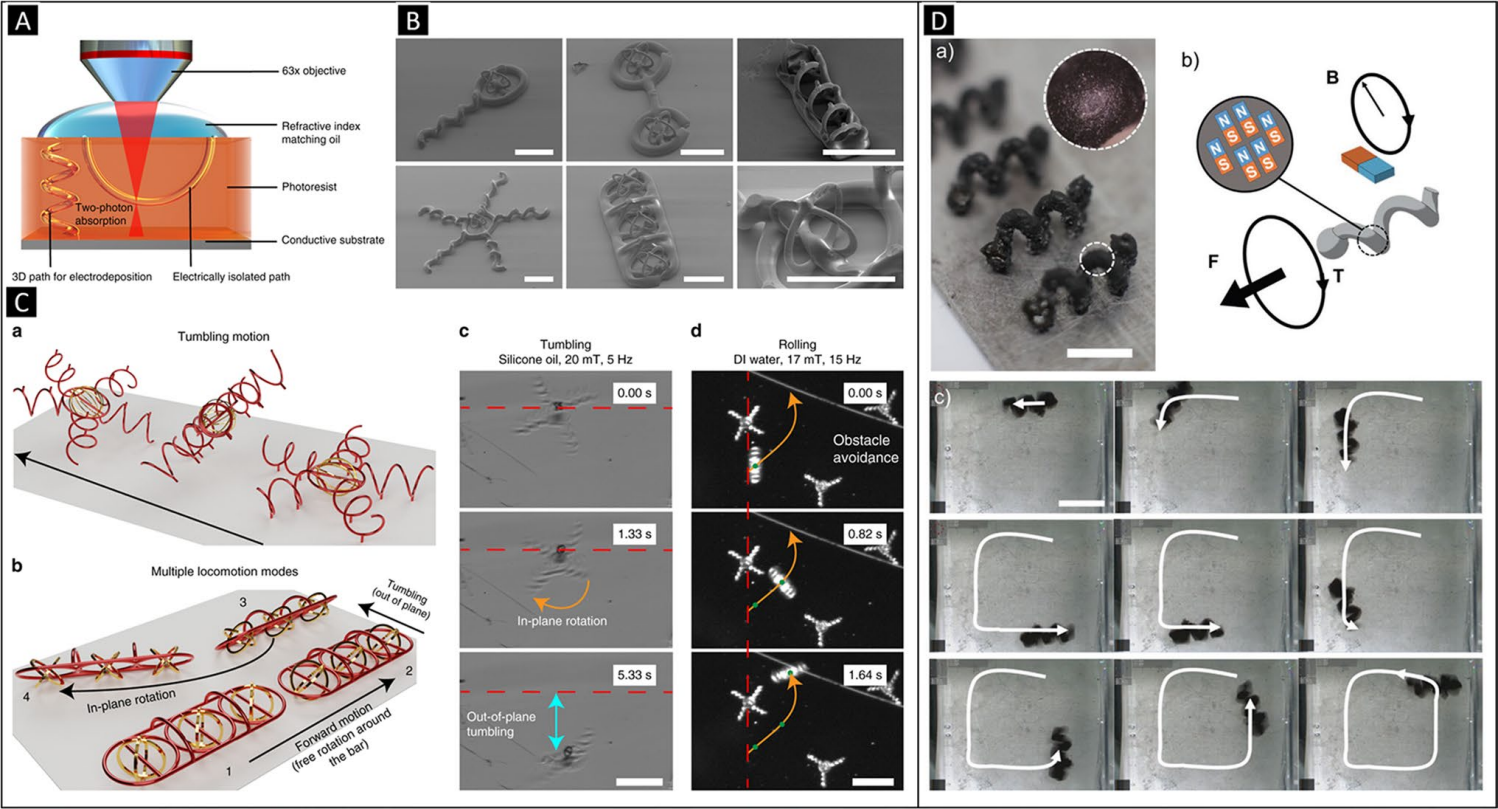
**A** Fabrication concept for mechanically interlocked multimaterial micromachines. Two types of microchannels are created in a photoresist deposited on a conductive substrate. In the picture, the helical microchannel enables the casted material to reach the conductive surface of the substrate, while the other curved microchannel does not enable the filling material to contact the substrate. The cavities allowing access to the substrate will enable depositing material by an electrochemical process, while the electrically isolated microchannels will be filled afterwards with water-soluble polymers. Finally, two dissimilar interlocked geometries composed of different materials (i.e., electroplated metal and casted polymer) can be generated after dissolving the mold. **B** SEM characterization of 3D mechanically interlocked architectures made of fully metallic Fe and PDMS parts (scale bar is 50 μm). **C** (a) Scheme of a spider-like microrobot comprising a cage-bar-ring architecture interlocked with five helical appendices. (b) A wheeled microrobot consisting of three spherical Fe microcages interlocked with an elongated polymer chassis. These structures can display forward motion and rolling along their short axis. (c) Out-of-plane tumbling and in-plane rotation of a spider-like microrobot (scale bar is 150 μm); (d) A wheeled microrobot rolling on a substrate and avoiding obstacles. (Figs. 7A, B, and C: Adapted with permission [17]. Copyright © 2020, Springer Nature.) **D** (a) Batch fabrication of 3D-printed sucrose-based BaFe12O19 composite helical millimeter-size robots (scale bar is 10 mm). The inset shows a magnified image of the surface. (b) Scheme and (c) manipulation of a pre-magnetized sugar-based magnetic helical robot corkscrewing under a rotating magnetic field of 30 mT at 5 Hz. (Adapted with permission [76]. Copyright © 2020, WILEY–VCH Verlag GmbH & Co. KGaA, Weinheim.)

Most magnetically propelled micro- and nanorobots have been made with ferromagnetic, ferrimagnetic or superparamagnetic materials, either in metal, alloy or polymer composite form. An exhaustive review has been recently published regarding this type of swimmers by Pumera, Zhang, and Pané [34••]. Here, we will briefly discuss some few recent achievements in the field, which we consider unprecedented in terms of their fabrication approach. For example, Magdanz et al. [29] have lately reported on the fabrication of sperm-template microrobots and the impact of the segmented magnetization on their propulsion. Electrostatic self-assembly of magnetic nanoparticles on nonmotile sperm cells was used to fabricate the hybrid microswimmers [31]. The conducted analysis on their motion under oscillating magnetic fields showed that the microrobots were able to swim by traveling waves. Yet, the shape of the generated waves was dependent on the magnetized cellular segments, subsequently affecting the fluid response and the propulsive thrust. Chen and co-workers have recently described a fabrication sequence comprising melt electrospinning, micromolding, and skiving in order to manufacture polycaprolactone/magnetite composite-based tadpole-like microrobots [73]. Figures 6 (E) and (F) show the fabrication scheme of these devices and the motion of the swimmer as a function of the rotating magnetic field. The microrobots were able to roll or propel. Transportation of several cargoes was also demonstrated. Choi and co-workers have developed 3D porous magnetic helical microrobots [74] (Fig. 6 (G)). The devices were fabricated by two-photon polymerization. Owing to the high surface area of the swimmers’ architecture, it was possible to load the device with drug. Additionally, ultrasound-assisted drug release was achieved thanks to the porosity structure of the swimmers’ chassis. By adjusting the acoustic field conditions, three modes of release were identified: natural, burst, and constant. Sitti and co-workers have recently reported on voxelated 3D small-scale magnetic soft machines by means of jig-assisted assembly [75]. Figure 6(H) shows a schematic of the process in which a 3D ring is created. Figure 6(H) also shows the manufacturing of the jigs, which contains permanent magnets to assist the assembly of the magnetic and nonmagnetic components. A ring and two half rings are sequentially plugged on the 3D jig to form the 3D ring. Pané and co-workers have demonstrated a template-assisted process to fabricate 3D micromachines consisting of mechanically interlocked magnetic hard 3D components made of electroplated iron with mold-casted 3D soft polymeric structures [17]. Figure 7(A) shows a schematic of the approach. Briefly, a photocurable polymer layer is deposited on a conductive substrate. Different 3D paths are then formed by direct laser writing. Those paths that reach the conductive substrate allow for electrodeposition of materials, while those that do not access the substrate allow for mold casting a polymer or another material. Figure 7(B) shows different mechanically interlocked metal–organic micromachines made of 3D parts of iron and different polymers. Multiple locomotion strategies such as tumbling or rolling with complex structures were possible as shown in Fig. 7 (C). Puigmartí, Pané, and co-workers have recently reported on the realization of millimeter-scale magnetic sugar-based helical swimmers by means of selective laser sintering [76]. Owing to the fast degradability of sugar structures, these devices could be used for rapid interventions in which a fast dissolution of the robot is needed. Figure 7(D) shows a batch of 3D-printed barium ferrite@sucrose helical swimmers and sequence of its motion under low rotating magnetic fields in silicon oil. Interestingly, the study also included a study on the mechanical properties of sugar-printed structures as a function of their level of caramelization.

## Conclusions

The field of small-scale robots is facing grand challenges for their practical use in several applications, especially in the area of biomedicine. A defying yet exciting endeavor is to identify suitable materials and fabrication schemes that can generate highly integrated micro- and nanorobots specifically designed for a set of tasks and fulfill all the requirements for a targeted application. Material science and chemistry have greatly contributed to narrow the challenges for the translation of small-scale robotic technologies from the bench to the real world. The extensive buffet of materials and fabrication approaches is there, and it is constantly growing. Now, the community of small-scale roboticists must concentrate their efforts in finding the most suitable applications.
